# Daily Mango Intake Improves Glycemic and Body Composition Outcomes in Adults with Prediabetes: A Randomized Controlled Study

**DOI:** 10.3390/foods14172971

**Published:** 2025-08-26

**Authors:** Raedeh Basiri, Kallie Dawkins, Saiful Singar, Lauren T. Ormsbee, Neda S. Akhavan, Robert C. Hickner, Bahram H. Arjmandi

**Affiliations:** 1 Department of Nutrition and Food Studies, George Mason University, Fairfax, VA 22030, USA; 2 Department of Health, Nutrition, and Food Sciences, Florida State University, Tallahassee, FL 32306, USArhickner@fsu.edu (R.C.H.); 3 Department of Kinesiology and Nutrition Sciences, University of Nevada Las Vegas, Las Vegas, NV 89154, USA; 4 Institute of Sports Sciences and Medicine, Florida State University, Tallahassee, FL 32306, USA

**Keywords:** prediabetes, glycemic control, blood glucose, body composition, mango

## Abstract

**Background:** Prediabetes is on the rise and can progress to type 2 diabetes and related cardiometabolic complications if left untreated. Nutrition plays a critical role in blood glucose regulation, and evaluating the effects of whole foods on indices of glycemic control and body composition within individuals with prediabetes is important. This study examined whether consuming one average fresh mango daily for 24 weeks could improve the blood glucose indices and body composition in individuals with prediabetes. **Methods:** The participants were randomly assigned to either a treatment group (~300 g mango daily for 24 weeks) or a control group (an isocaloric granola bar daily for 24 weeks). Laboratory visits were conducted at baseline and at weeks 6, 12, and 24 to distribute the study regimen and collect anthropometric measurements, body composition data, and blood samples. **Results:** Twenty-three participants completed this study (treatment: *n* = 11; control: *n* = 12). At week 24, the treatment group had lower fasting blood glucose (*p* < 0.02) and improved insulin sensitivity (QUICKI, *p* = 0.02), and indicated a trend toward lower insulin resistance (HOMA-IR, *p* = 0.05) compared with the control. The mean HbA_1_c remained stable in the treatment group but increased in the control group (*p* = 0.02). In the treatment group, the body fat percentage showed a borderline decrease (*p* = 0.05), while the fat-free mass increased (*p* < 0.03); no significant changes were observed in the control group. **Conclusions:** The daily consumption of mango for 24 weeks improved the glycemic control, insulin sensitivity, and body composition in adults with prediabetes, which supports the potential of mango as a practical dietary intervention for metabolic health.

## 1. Introduction

According to the Centers for Disease Control and Prevention (CDC), more than 130 million adults in the United States are currently living with diabetes or prediabetes, including an estimated 98 million individuals with prediabetes—a condition that, without timely management, can progress to type 2 diabetes, heart disease, and stroke [1]. Projections indicate that, by 2030, nearly 40% of U.S. adults will be affected by prediabetes [2]. Early identification and prevention during the prediabetic stage are critical, as many individuals remain unaware of their condition until symptoms or complications arise [3].

Nutrition plays a vital role in glycemic control, and healthy eating supports both improved blood glucose management and long-term overall health [4,5,6]. In the Diabetes Prevention Program, lifestyle intervention reduced the incidence of type 2 diabetes by 58%, whereas metformin reduced it by 31% compared with placebo [7]. As such, identifying accessible, natural strategies to mitigate these risk factors may be key to delaying the onset of diabetes and reducing associated complications [8,9,10,11,12]. Dietary interventions that are rich in fruits, vegetables, and bioactive compounds have demonstrated positive effects on glycemic control and metabolic health [13,14]. Mango (*Mangifera indica* L.) is a tropical fruit that contains several bioactive compounds—including carotenoids, ascorbic acid, dietary fiber, phenolic compounds, gallic acid, and quercetin—that may contribute to its potential health benefits [15]. Preclinical studies have shown that freeze-dried mango supplementation can positively influence the body composition and improve the glucose and lipid profiles in mice fed a high-fat diet [16]. Evidence from human studies on mango and glycemic outcomes is mixed. Acute crossover trials report lower postprandial glucose and insulin levels with mango vs. isocaloric refined snacks, with greater satiety, suggesting short-term benefits of fruit matrix and fiber on glycemic excursions [17,18]. By contrast, longer interventions show improvements in some, but not all, glycemic markers: a 12-week crossover RCT lowered patients’ fasting glucose and inflammation without changes in insulin or HbA1c [19], an 8-week trial reduced patients’ 2 h OGTT glucose without weight loss [20], and a 12-week pilot study lowered patients’ fasting glucose yet did not improve their HbA1c or HOMA-IR and even increased the insulin level among men [21].

Despite these promising results, no controlled, long-term studies have directly assessed how daily mango consumption affects both the short- and long-term glycemic control and body composition in individuals with prediabetes. Therefore, the aim of this study was to evaluate the effects of 24 weeks of daily mango consumption on indices of glycemic control and body composition in this at-risk population. We hypothesized that daily consumption of one average fresh mango (Tommy Atkins, approximately ~500 g with an edible portion of about 300 g) would reduce fasting plasma glucose and insulin resistance, while improving body composition indices, which could further support better glucose management.

## 2. Materials and Methods

This study was conducted at Florida State University, was approved by the Florida State University Institutional Review Board (IRB Study ID: STUDY00002545), and was registered at Clinicaltrials.gov (NCT05571800). Independent-living adult men and postmenopausal women between the ages of 50 and 70 years, with a body mass index (BMI) between 18.5 kg/m^2^ and 34.9 kg/m^2^ and fasting blood glucose levels ranging from 100 to 125 mg/dL or HbA1c between 5.7 to 6.4% were included in this study. Exclusion criteria included a diagnosis of diabetes, cardiovascular disease, uncontrolled hypertension (≥160/100 mmHg), active chronic diseases (e.g., cancer, thyroid, kidney, liver, or pancreatic disease), hormone replacement therapy, adherence to any specific diet, smoking, or heavy alcohol consumption (>12 alcoholic drinks per week). Individuals who reported frequent mango consumption (more than five mangoes in the past two weeks) were excluded. We excluded participants who reported frequent mango consumption to reduce potential adaptation effects. Habitual intake of a specific fruit can alter glycemic and appetite responses via behavioral conditioning and physiological changes, including fiber tolerance, gastric emptying dynamics, and gut-microbiota-mediated metabolism of fruit constituents, which may attenuate or exaggerate the intervention effect and introduce expectancy/palatability bias [22,23,24,25,26,27]. Excluding frequent consumers helped standardize exposure and improve internal validity.

A sample size of 24 participants was determined to provide >80% power to detect significant differences in primary outcomes (*p* < 0.05). The sample size was calculated using G*Power (version 3.0.10) for a two-group repeated-measures design with four time points, assuming an alpha of 0.05, a power of 80%, and an effect size of 0.25 for changes in fasting blood glucose, which is considered a clinically meaningful outcome [28].

### 2.1. Screening, Enrollment, and Randomization

Volunteers were recruited using community-based strategies in the greater Tallahassee area, including printed flyers, social media, and emails. Screening and baseline assessments were conducted across two visits. At the screening visit, a member of the research team collected fasting blood glucose, HbA1c, blood pressure, and anthropometric measurements at the Sandels Building Clinical Area, Florida State University (FSU). Participants who met the eligibility criteria were provided with verbal and written explanations of this study and were given opportunities to ask questions. Participants then signed an informed consent form, and a copy of the signed consent form was provided to them.

Participants (*n* = 24) were randomized into treatment and control groups using GraphPad QuickCalcs. The treatment group received approximately 300 g of fresh mango daily, while the control group received a calorie-matched granola bar each day. The dose was chosen to represent a practical, single-portion serving that fits within daily fruit recommendations, delivers adequate fiber and bioactive compounds to test our hypothesis on glycemic regulation and body composition, and is consistent with prior mango trials while supporting adherence and gastrointestinal tolerance [20]. Participants in the control group consumed a commercially available granola bar that was isocaloric to the mango portion and intended for the same snacking occasion. We selected the bar to (1) reflect a typical snack in free-living settings, (2) standardize composition to improve feasibility and adherence, and (3) provide a calorie-matched comparator with lower fruit-specific bioactive content, allowing evaluation of mango’s whole-fruit matrix effects beyond energy and sugar content alone. Table 1 presents a comparison of the nutrient composition between a Tommy Atkins mango and the granola bar [29,30].

We chose to compare the effects of consuming one average mango (~300 g edible portion) with those of a calorie-matched popular snack in the United States to isolate the unique impact of whole fruit, as a healthy carbohydrate source, on postprandial glycemia and body composition. By matching the calorie content between groups, we controlled for energy load and instead assessed whether the intrinsic matrix of mango—rich in fiber, vitamins, and polyphenols [31]—would yield more favorable glycemic and body composition responses compared to a processed snack.

### 2.2. Baseline Visit

At the baseline visit, a 20 mL venous blood sample was collected from participants in a fasted state, and anthropometric and body composition measurements were conducted. Each participant received their first 6-week supply of their assigned regimen. Participants were instructed to maintain their habitual diets and refrain from intentionally changing their body weight or physical activity levels during the study period. A compliance calendar was provided to each participant to track their daily intake and record any missed or leftover portions at baseline and 6, 12, and 24-week visits. Compliance was defined as ≥80% adherence to the assigned regimen; participants falling below this threshold were considered noncompliant and removed from analysis. Follow-up appointments at weeks 6, 12, and 24 were scheduled, and participants were notified via phone and email.

### 2.3. Anthropometric Measurements

Height, weight, and waist and hip circumferences were measured at baseline, and at 6, 12, and 24 weeks, following the WHO STEPS protocol [32]. Height was measured without shoes using a stadiometer; weight was measured using a digital scale (Seca, Chino, CA, USA). Circumferences were measured using a Gulick fiberglass measuring tape with a tension handle (Creative Health Products, Ann Arbor, MI, USA).

### 2.4. Body Composition Analysis

Body composition was assessed using ImpediMed SFB7 BIS (ImpediMed Limited, Pinkenba, QLD, Australia), which measured fat mass, lean mass, and total body water. The device applied a small alternating current through the body to measure electrical impedance, which varies between tissues based on their water and electrolyte content.

### 2.5. Finger Stick Blood Testing

Fingerstick blood samples were collected at the screening visit to assess fasting blood glucose using a OneTouch^®^ (LifeScan, Malvern, PA, USA) glucometer. Hemoglobin A1c (HbA1c) was measured at baseline, 12 weeks, and 24 weeks using the A1cNow^®^ system (PTS Diagnostics, Whitestown, IN, USA). All measurements were performed using whole blood samples and conducted immediately after collection, without storage.

### 2.6. Blood Collection and Biochemical Assessments

Fasting venous blood samples (20 mL) were collected at baseline and 6-, 12-, and 24-week visits. Samples were centrifuged within one hour at 1500× *g* for 15 min at 4 °C [33]. Plasma was separated, aliquoted, and stored at −80 °C until analysis. Fasting plasma glucose was measured using Piccolo Xpress Chemistry Analyzer (ABAXIS, Union City, CA, USA), and insulin was measured using ELISA (R&D Systems, Minneapolis, MN, USA).

### 2.7. Calculating HOMA-IR

Insulin resistance was estimated using homeostatic model assessment of insulin resistance (HOMA-IR), a commonly employed and validated tool in clinical studies. The HOMA-IR value was calculated using the following formula [34]:

HOMA-IR = [Fasting blood glucose (mg/dL) × Fasting insulin (µU/mL)] ÷ 405

### 2.8. Assessment of Insulin Sensitivity Using QUICKI

Quantitative Insulin Sensitivity Check Index (QUICKI) was used to estimate insulin sensitivity based on fasting plasma glucose and insulin levels. This index is a validated surrogate measure that shows a strong correlation with the hyperinsulinemic-euglycemic clamp, the gold standard for assessing insulin sensitivity [34,35]. QUICKI was calculated using the following equation:

QUICKI = 1 ÷ [log (fasting insulin µU/mL) + log (fasting glucose mg/dL)]

### 2.9. Statistical Analyses

Baseline demographic characteristics were compared between treatment and control groups using independent *t*-tests for continuous variables (age, BMI, waist–hip ratio) and chi-square tests for categorical variables (sex, race, ethnicity). Variables that showed significant between-group differences (*p* < 0.05) and exhibited significant interactions with the outcome variable were included as covariates in subsequent models. To evaluate treatment effects over time, repeated measures analysis of the general linear model was conducted. When significant main effects or interactions were identified, post hoc multiple comparison tests were applied. All statistical analyses were conducted using SPSS (IBM SPSS Statistics for Windows, Version 29.0). A *p*-value of <0.05 was considered statistically significant for all analyses.

## 3. Results

A total of 23 participants completed this study (treatment: *n* = 11; control: *n* = 12). Baseline comparisons of key demographic and clinical characteristics showed no significant differences in age or waist–hip ratio between the groups; however, the BMI differences approached significance (*p* = 0.09). Chi-square tests indicated significant differences in the distribution of sex, race, and ethnicity (*p* < 0.01 for all), suggesting baseline imbalance in these variables. Therefore, interactions between sex, race, ethnicity, and BMI were assessed for each outcome, and those with significant interactions were included as covariates in the analytical models. Table 2 presents the baseline demographic characteristics of the participants.

### 3.1. Changes in Fasting Blood Glucose and HbA1c

There were no significant interactions between the fasting blood glucose or HbA1c and sex, race, ethnicity, or baseline BMI; thus, these covariates were excluded from the analytical models. At baseline, the mean ± SE for fasting blood glucose was 113.3 ± 2.7 mg/dL in the treatment group and 116.5 ± 2.6 mg/dL in the control group, with no statistically significant difference being observed between the groups. Over the study period, the mean ± SE for fasting glucose numerically decreased to 107.0 ± 5.1 mg/dL in the treatment group but marginally increased to 125.3 ± 4.8 mg/dL (*p* = 0.07) in the control group. At 24 weeks, the treatment group had lower fasting glucose than the control group (mean difference ± SE: 18.3 ± 7.0 mg/dL, *p* < 0.02).

The mean ± SE for the HbA1c at baseline was slightly higher in the treatment group compared to the control group (5.7 ± 0.2% vs. 5.5 ± 0.2%), although this difference was not statistically significant. The mean HbA1c remained the same in the treatment group during the 24 weeks of follow-up, but increased to 5.9 ± 0.1% in the control group (*p* = 0.02)

### 3.2. HOMA-IR and QUICKI

The baseline mean ± SE for the HOMA-IR was lower in the treatment group compared to the control group (2.1 ± 0.6 vs. 3.1 ± 0.6), but the difference was not statistically significant. Over the 24-week period, the mean HOMA-IR decreased slightly in the treatment group (from 2.1 to 2.0) but increased in the control group (from 3.1 to 3.6), which resulted in a between-group difference at week 24 (*p* = 0.05).

**Figure 1:**
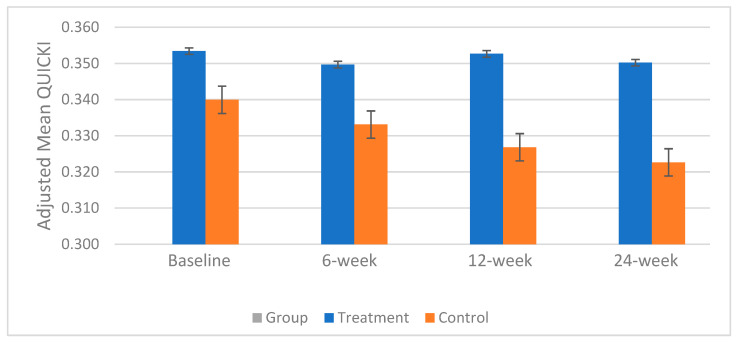
Changes in mean QUICKI over the 24-week study period. Participants visited the lab at baseline and weeks 6, 12, and 24. A statistically significant difference between groups was observed at week 24 (*p* < 0.02). Error bars represent standard errors of the mean (SEM).

The mean ± SE QUICKI at baseline was slightly higher in the treatment group compared to the control group (0.35 ± 0.01 vs. 0.34 ± 0.01); however, the difference was not statistically significant. Race demonstrated a significant interaction with the QUICKI and was therefore included as a covariate in the analysis. Throughout this study, the mean QUICKI remained almost stable in the treatment group, while it declined in the control group (from 0.34 ± 0.01 to 0.32 ± 0.01), which resulted in a statistically significant difference between the groups at week 24 (*p* < 0.02). The adjusted mean QUICKI changes over the study period for both groups are shown in Figure 1.

### 3.3. Body Composition, BMI, and Waist-to-Hip Ratio

The mean ± SE body fat percentage in the treatment group decreased from 30.5 ± 2.0% to 29.1 ± 2.0%, while in the control group it went from 31.5 ± 2.0% to 30.6 ± 2.0%. This reduction in the treatment group trended toward statistical significance by week 12 (*p* = 0.05), whereas no significant change occurred in the control group.

Race and sex showed significant interactions with fat-free mass and were included as covariates in the analysis. In the treatment group, the fat-free mass declined from 71.4 ± 1.7% to 65.6 ± 3.3% by week 6 (*p* < 0.05) but increased to 72.5 ± 1.8% by week 24 (*p* < 0.03). No significant changes were noted in the control group. The between-group difference at week 24 approached statistical significance (mean difference ± SE = 5.43 ± 0.06, *p* = 0.06). The mean ± SE total body water increased from 50.9 ± 1.5% to 51.8 ± 1.5% in the treatment group and from 50.2 ± 1.5% to 50.8 ± 1.5% in the control group; however, these changes were not statistically significant.

In the treatment group, the mean ± SE BMI marginally decreased from 24.5 ± 1.3 to 24.1 ± 1.4 kg/m^2^ by week 12 (*p* = 0.07), but returned to 24.5 ± 1.4 kg/m^2^ by week 24. In contrast, the control group showed a consistent numerical increase in BMI from 28.6 ± 1.3 kg/m^2^ at baseline to 29.0 ± 1.4 kg/m^2^ at week 24.

**Figure 2:**
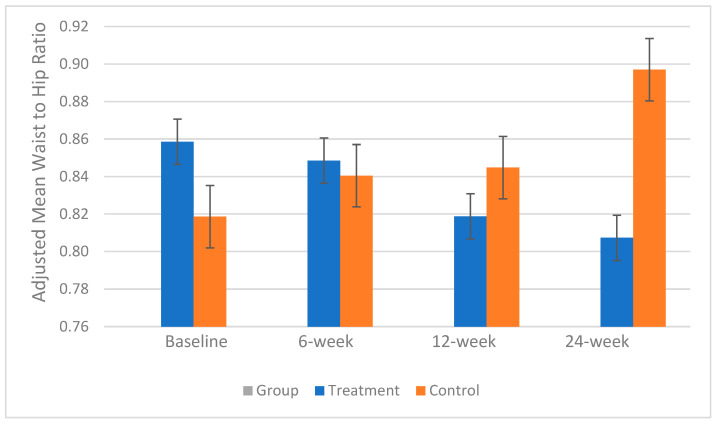
Changes in mean waist–hip ratio over the 24-week study period. Participants visited the lab at baseline and weeks 6, 12, and 24. Error bars represent standard errors of the mean (SEM).

A significant interaction between the baseline BMI and waist–hip ratio was observed, and this variable was therefore included as a covariate in the analysis. The mean ± SE waist–hip ratio was not significantly different between groups throughout the study. However, in the treatment group, it decreased from 0.86 ± 0.04 to 0.82 ± 0.03 (*p* < 0.03) by week 12. In contrast, the control group experienced a consistent and significant increase from 0.82 ± 0.04 to 0.90 ± 0.03 over the study period (*p* = 0.01), which may have clinical relevance. The changes in the adjusted mean waist–hip ratio are presented in Figure 2.

## 4. Discussion

Our findings support and expand emerging evidence on the metabolic benefits of daily fresh mango consumption in individuals with prediabetes. The participants who consumed one average mango daily for 24 weeks experienced lower fasting blood glucose levels compared to the control group (*p* < 0.02). Additionally, the mean HbA_1_c levels remained stable in the treatment group, while they increased significantly in the control group. These glycemic improvements were accompanied by lower insulin resistance, as measured by the HOMA-IR (*p* = 0.05), and greater insulin sensitivity, as measured by the QUICKI (*p* < 0.02). These results are in line with those of Pett et al., who reported improved insulin sensitivity and a reduced HOMA-IR following a four-week mango intervention in overweight or obese adults with low-grade inflammation [36]. The longer duration of our study (24 weeks) suggests that the beneficial effects of mango on glycemic control may be both early onset and sustained over time.

In terms of body composition, the participants in the treatment group maintained their BMI over 24 weeks, while those in the control group experienced a numerical increase. A crossover trial by Rosas et al. found that 12 weeks of daily mango intake improved blood glucose but had no significant effects on body composition [19]. Our study demonstrated not only improved glycemic measures but also favorable changes in body composition, including a reduction in body fat percentage (*p* = 0.05) and a significant increase in fat-free mass (*p* < 0.03) in the treatment group. Importantly, there were no significant changes in total body water, which suggests that the observed increase in fat-free mass likely occurred independently of fluid retention. The longer intervention period in our study (24 weeks vs. 12 weeks) may explain the more pronounced effects compared to the Rosas et al. study. Another potential reason might be the differences between the populations of the studies, as it has been shown that individuals respond differently to different foods, likely due to interindividual variability. A recent study using transcriptomic-based clustering suggested that mango’s impact on metabolic health may depend on individual gene expression patterns [37]. Our findings are also supported by preclinical data. A study using a high-fat diet mouse model showed that mice on a high-fat diet that received freeze-dried mango pulp had significantly lower total body fat and epididymal fat mass, with efficacy comparable to pharmaceutical agents like fenofibrate and rosiglitazone [16]. These results parallel our human data and support the potential of mango as a functional food for improving glucose metabolism and body composition.

The observed changes in body composition are clinically important, given the strong links between body composition and type 2 diabetes risk demonstrated in prior studies. Haines and colleagues (2022) examined young adults under 50 and found that a lower skeletal muscle mass was independently associated with higher type 2 diabetes prevalence, even after adjusting for body fat distribution [38]. Similarly, another study reported that decreases in appendicular skeletal muscle mass index (pASMMI) and lean body mass index (pLBMI) scores over time correlated with new-onset diabetes in overweight and obese adults, indicating that preserving muscle mass helps prevent diabetes development [39]. Additionally, it has been shown that a greater relative muscle mass was inversely associated with insulin resistance and risk of prediabetes across a large U.S. population sample, which underscores muscle mass as a modifiable protective factor against glycemic deterioration [40]. Moreover, it has been shown that those with a high waist–hip ratio had 1.56 times the odds (95% CI 1.18–2.07), those with high body fat percentage had 1.62 times the odds (95% CI 1.01–2.68), and those with larger visceral fat area had 1% higher odds per cm^2^ (OR 1.01; 95% CI 1.01–1.02) of developing type 2 diabetes [41]. These findings are consistent with the hypothesis that greater muscle mass may be protective and excess adiposity may contribute to the risk of type 2 diabetes; however, causal mechanisms cannot be inferred from this study.

Our findings align with evidence from systematic reviews/meta-analyses that whole-fruit interventions can modestly improve glycemic control, most consistently fasting glucose, with the effects varying by fruit type [22]. Some data also suggest larger benefits in prediabetes than in established diabetes, underscoring the positive impact of early intervention [42]. Emerging evidence further indicates that substituting free/added sugars with intrinsic sugars is associated with more favorable adiposity profiles, supporting a “sugar source and matrix” perspective rather than a sugar-grams-only view [43,44]. Although mango was our specific test food, our findings are consistent with studies on other fruits and may extend to a broader class of plant functional foods. Despite containing more intrinsic sugars than the isocaloric granola-bar comparator (≈32.1 g vs. 11 g), mango produced more favorable glycemic indices and body composition changes. These results support a matrix-based, rather than sugar-only, framework for dietary guidance. Practically, this suggests that replacing refined snacks with whole fruits in the diets of individuals at risk for diabetes may offer metabolic and other health benefits. Future studies should evaluate additional fruit types and forms, assess dose–response relationships, and incorporate dietary biomarkers to clarify mechanisms and broaden applicability.

A major strength of our study is its 24-week duration, which enabled us to evaluate both short- and longer-term effects of fresh mango consumption. However, there are limitations that need to be considered. The statistical power of this study was calculated for the primary outcome (fasting blood glucose) only. Our analyses of other endpoints were exploratory and not adjusted for multiple comparisons. The limited racial and ethnic diversity of our sample may reduce the generalizability of the findings. Although the distribution of sex, race, and ethnicity was different between the groups at baseline, we adjusted for these variables by including them as covariates in all relevant models. While this approach helps account for baseline differences, the possibility of residual confounding remains, and is a common challenge in clinical trials. Objective dietary measures were not evaluated in this study. However, because both groups self-reported their intake, any inaccuracies likely affected them equally, which may attenuate between-group differences.

Future studies should incorporate objective dietary biomarkers (e.g., plasma carotenoids, urinary polyphenols) to improve their accuracy and validity. Additionally, they should include more diverse populations and evaluate the mechanisms underlying the observed effects, such as the role of mango’s bioactive compounds in modulating insulin signaling and inflammation. Longer follow-up periods and comparisons with other fruits or dietary strategies may also help clarify mango’s unique benefits to metabolic health. Lastly, future studies should explore the broader health benefits of mango consumption beyond glycemic control and body composition, including potential metabolic pathway effects and gene expression changes, given that individuals may respond differently to the same foods.

## 5. Conclusions

In conclusion, daily consumption of mango for 24 weeks improved both short- and long-term blood glucose control, reduced insulin resistance, enhanced insulin sensitivity, and promoted favorable changes in body composition among individuals with prediabetes. These findings suggest that incorporating fresh mango into the diet may offer a practical, food-based strategy to support glycemic control and improve the body composition in individuals at high risk for type 2 diabetes.

## Figures and Tables

**Table 1:** Nutritional comparison of fresh mango and calorie-matched granola bar.

Nutrient	Tommy Atkins Mango (~300 g Edible)	Granola Bar (2 bar, 22 g)
Calories (kcal)	195	190
Total Fat (g)	1.7	7
Saturated Fat (g)	0.0	1
Sodium (mg)	<7.5	140
Total Carbohydrate (g)	45.9	29
Dietary Fiber (g)	5.4	2
Total Sugars (g)	32.1 ^2^	11 ^1^
Protein (g)	1.7	3
Iron (mg)	<0.75	1
Vitamin C (mg)	30.6	0.0

^1^ Added sugar; ^2^ natural sugar.

**Table 2:** Demographic characteristics of participants at baseline.

Variable	Treatment (Mean ± SD)	Control (Mean ± SD)	*p*-Value
Age (years)	66.18 ± 3.25	65.17 ± 4.93	0.60
Sex (Female/male)	10/1	7/5	<0.01
BMI ^1^ (kg/m^2^)	24.46 ± 3.26	28.46 ± 5.04	0.09
Waist-to-Hip Ratio	0.85 ± 0.12	0.83 ± 0.13	0.40
Fasting Blood Glucose (mg/dL)	113.27 ± 7.16	116.5 ± 10.13	0.40
Race (White/black)	10/1	10/2	<0.01
Ethnicity (Hispanic/non-Hispanic)	1/10	0/12	<0.01

^1^ Body mass index.

## Data Availability

The original contributions presented in the study are included in the article, further inquiries can be directed to the corresponding author.
